# In vivo evidence of h*tid *suppressive activity on ErbB-2 in breast cancers over expressing the receptor

**DOI:** 10.1186/1479-5876-8-58

**Published:** 2010-06-17

**Authors:** Ursula Kurzik-Dumke, Manuela Hörner, Maria R Nicotra, Michael Koslowski, Pier G Natali

**Affiliations:** 1 Institute of Medical Microbiology and Hygiene, Comparative Tumor Biology Group, University Medical Center, Johannes Gutenberg University, Obere Zahlbacher Str. 63, 55131 Mainz, Germany; 2 Inst. Molecular Biology and Pathology CNR, Rome, Italy; 3 Experimental and Translational Oncology III, University Medical Center, Johannes Gutenberg University, Obere Zahlbacher Str. 63, 55131 Mainz, Germany; 4 Immunology Laboratory, "Regina Elena" National Cancer Institute, Via delle Messi d'Oro 156, 0158 Rome and CIMBO Laboratories, "G.d'Annunzio" University, Chieti, Italy

## Abstract

### Background

H*tid *encoded proteins are physiological partners of a wide spectrum of molecules relevant to neoplastic transformation. One of the molecular ligands of the cytosolic hTid-L and hTid-I forms is the ErbB-2 receptor variably over expressed in diverse solid tumors. Altered ErbB-2 signalling is associated with an unfavourable prognosis in about 30% of human breast malignancies.

### Methods

We evaluated h*tid *and *HER-2 *expression by quantitative real time PCR in tumors of different TNMG status and by immunohistochemistry in a cohort of breast tumors of the Luminal A, B, HER-2 and triple negative subtype.

### Results

The RT-PCR analysis revealed that aberrant expression of all three h*tid *forms correlates with malignant transformation. Furthermore, elevated hTid-L expression can be associated with less aggressive tumors. The immunohistochemical testing revealed that tumors of the luminal A subtype are characterized by a high level of h*tid *(81%). In contrast h*tid *expression is significantly lower in tumors of the Luminal B (20%) and HER-2 (18%) subtype over expressing the receptor and in the triple negative (40%) more aggressive malignancies. A statistically significant inverse correlation between h*tid *and ErbB-2 expression was found in human breast (p < 0,0001) and non-mammary tumors (p < 0,007), and in transgenic mice carrying the rat *HER-2/neu *oncogene.

### Conclusions

Our findings provide in vivo evidence that h*tid *is a tissue independent and evolutionarily conserved suppressor of ErbB-2.

## Background

H*tid-*1 is the human counterpart [1] of the *Drosophila melanogaster lethal(2)tumorous imaginal discs *(*l(2)tid*) tumor suppressor gene [2]. (In the following we omit the number "1" in the designation of the gene because it suggests the presence of a further copy in the genome. As sequencing of the human, mouse and *Drosophila *genomes revealed only one *tid *gene is present in all these species.)

The *tid *genes encode members of the highly conserved DNAJA3 protein family acting as Hsp70/Hsc70 co-chaperones [3-5]. In the fly, defined recessive mutations have been shown to be oncogenic in cell lineages responsive to the signalling mediated by Patched (Ptc), the receptor for the Hedgehog (Hh) signalling molecule [6]. The physiological Tid-Ptc binding is conserved in the fly, in the mouse and in man [6,7]. Using the yeast two-hybrid technique h*tid *has been isolated as a molecular partner of a variety of tumor related proteins [6-15] including receptor tyrosine kinases (RTK) such as TRK-A [14] and ErbB-2 [15] of the epidermal growth factor receptor (EGFR) family comprising four receptors (ErbB1-4) [16]. As for other members of these receptors, the ErbB-2 signal output is controlled by a dynamic equilibrium between the on/off states. In this regard, in vitro studies have shown that interaction of the cytosolic hTid-L and hTid-I proteins [1,7] with ErbB-2 promotes ubiquitination and degradation of the receptor resulting in down regulation of its signalling thus, of its oncogenic potential/activities [15]. (The aforementioned hTid-I form, 453 amino acids in size [1,7] is designated by Kim *et al. *[15] as hTid-S.). This is of interest since over expression of ErbB-2, often caused by gene amplification, characterizes 20-30% of human breast cancers being casually linked to an aggressive clinical course of these tumors [17] and a variable percentage of extra-mammary tumors [18]. These data are consistent with the biology of the EGFR family members mediating control of cellular responses such as proliferation, differentiation, survival and apoptosis, essential for the maintenance of the transformed and metastatic phenotype. Thus, in the context of the functional link between the h*tid *encoded proteins and ErbB-2 in the present study we addressed the question whether in human sporadic breast tumors the in vivo expression profiles of the two tumor genes provide support for h*tid *function as a negative regulator of ErbB-2 activity. Since h*tid *encodes three splice forms [1,7] we compared their expression levels with that of the *HER*-2 transcript using quantitative real-time PCR (RT-PCR) and the comparative Ct method for relative quantification [19] in a panel of human breast tumors of diverse differentiation grades [20,21]. Furthermore, we performed an extensive phenotypic comparative analysis of the expression of the two target genes in a panel of human breast cancers classified according to Sotiriou and Pusztai [22] as the subtypes luminal A, luminal B, HER-2 and triple negative, and by non breast tumors over expressing ErbB-2. Furthermore, since the identification of an experimental in vivo model mimicking human breast tumors is of great interest to dissect the oncosuppressive activity of the *tid *gene at the molecular level, we investigated mammary malignancies in transgenic mice carrying the rat counterpart of *HER-*2/*neu*. The data gained by RT-PCR showed that the single h*tid *splice forms [1,7] are differentially expressed in normal mammary epithelium. Whereas the h*tid*-I form shows the highest expression level (2^-ΔCt ^= 0,50), the h*tid*-L form expression level is about 7 times lower (2^-ΔCt ^= 0,07). The h*tid*-S form is present only residually (2^-ΔCt ^= 3,18 × 10^-5^). Generally, the expression level of each of the single h*tid *splice forms is lower as that determined for *HER*-2 (2^-ΔCt ^= 4,99). In breast tumors the expression levels of all three h*tid *transcripts are altered. As a result the amount-ratio among the diverse h*tid *forms and the *HER-2 *transcript changes drastically.

The phenotypic analysis of tumor specimens demonstrated that hTid and ErbB-2 expression are inversely correlated in breast cancer either primary (p < 0.0001) or metastatic (p < 0.023), in primary non-breast tumors (p < 0,007) and in breast tumors generated in *HER-2/neu *transgenic mice. Overall these results identify h*tid *as a novel negative modulator of *HER-2*, suggesting that strategies capable of increasing or stabilizing its cellular level may result into a decrease of the oncogenic signalling mediated by the receptor.

## Materials and methods

### Patients and tissues

The specimens of breast and non breast tumors employed in this study originate from patients free from therapy undergoing treatment at various institutions. Normal breast tissue was obtained from cosmetic mammoplast. Samples used for RT-PCR originate from archive of tumor samples kindly provided by Dr. Ö. Türeci of the Experimental and Translational Oncology III, University Medical Center, Johannes Gutenberg University, Mainz. Specimens assayed by immunohistochemistry were obtained at the "Regina Elena" Cancer Institute, Rome, Italy. The patients consented to the experimental use of the specimens as requested by the Institutional Ethical Committee (IRE resolution of n° 7 of July 2^nd^, 2003 adjourned on January,1^st^, 2006). All tumor samples used in this study were characterized with respect to their TNMG status according to WHO specifications following the criteria of the International Union Against Cancer UICC [20] and the Elston and Ellis method [21]. Freshly collected tissue samples were divided into two parts. One part was processed for routine histopathological examination, the other was immediately shock frozen. The collected samples were stained with 1% toluidine blue to monitor morphology and the ratio of glandular epithelium and interstitium. Exclusively samples characterized by high amount of glandular epithelium, over 80%, were used for RT-PCR and immunohistochemistry. Non-consecutive 4 μm thick cryostat sections were prepared, fixed for 10 min in absolute acetone, and either submitted to indirect immunoperoxidase (IPP) staining or stored at -20°C over a period of a month with no loss of immune reactivity. The immunohistochemical analysis was performed on 75 randomly selected cases of infiltrating ductal carcinomas and 30 metastatic (25 lymphathic and 5 extra-lymphatic lesions) carcinomas building the first cohort. The second cohort of specimens consisted of 58 primary infiltrating breast tumors classified according to Sotiriou and Pusztaim [22] into the four distinct subtypes: luminal A, luminal B, HER-2 and triple negative on the basis of the results of immunohistochemical evaluation of expression of the estrogen and progesterone hormone receptors and the ErbB-2 receptor kinase, cytokeratins and the proliferation marker Ki67. This cohort encompassed a total of 24 tumors over expressing ErbB-2 (7 cases classified as luminal B and 17 cases of the HER-2 subtype), and a total of 34 ErbB-2 negative tumors (8 luminal B, 16 luminal A cases and 10 triple negative tumors). Non breast tumors were represented by a selected panel of 18 malignancies including thyroid, colon, ovarian and renal carcinomas. Experimental breast tumors developed during a period of 22 and 30 weeks in transgenic Balb/c mice carrying the rat *HER-2/neu *oncogene [23] were kindly provided by Prof. Guido Forni (Dept. of Clinical and Biological Sciences, Univ. of Turin, Italy) and processed like the human specimens.

### Antisera

Rabbit anti-hTid antiserum recognizing both the human and the mouse Tid proteins was generated, purified and characterized as previously described [6,7,13]. The rabbit polyclonal antiserum (AO 485) to human ErbB-2 cross reacting with the rat receptor was obtained from DAKO Cytomation (Denmark). The polyclonal antibody raised in chickens (GTX14027) recognizing the rat receptor was obtained from Gene Tex, Inc. (S. Antonio, TX. USA). Alexa fluor 594-labelled goat anti-rabbit IgG antiserum was purchased from Molecular Probes (Eugene, OR. USA). Fluorescein (FITC)-labelled rabbit anti chicken IgG was purchased from Sigma (St. Louis MA, USA).

### Immunohistochemical staining

Indirect immunoperoxidase staining of frozen tissue sections was performed according to standard procedures using the Vectastain ABC kit (Vector Labor, Burlingame, CA). Anti-hTid [6,7,13] was used at a concentration of 10 μg/ml while the anti ErbB-2 antibody (AO485) was employed as suggested by the manufacturer. Sections were counterstained using Mayer's hematoxylin. Positive controls were represented by archival specimens of known expression of both antigens, while negative controls were represented by sections incubated with normal rabbit immunoglobulins or isotype matched mouse imnunoglobulins. The evaluation of the staining was performed as follows: h*tid *was scored negative when cytoplasmic staining was absent or very weak and positive when a homogeneous granular cytoplasmic staining, ranging from moderate (+) to strong (2+/3+), was detected. ErbB-2 expression was established on the basis of a strong 3+ cell membrane staining or a 2+ staining intensity with a positive FISH assay [24]. Scoring of the expression of the antigens investigated was performed independently by two pathologists. Double immunofluorescence staining of breast tumors developed in *HER-2/neu *transgenic mice was performed by incubating the tumor sections at first with the rabbit anti-hTid antiserum and Texas Red labelled anti rabbit IgG. After blocking with decomplemented normal rabbit serum at a dilution of 1:100, 30 min, the sections were stained with chicken anti rat HER-2 antibody and the FITC-labelled rabbit anti chicken IgG. Sections were evaluated employing a Leica DMIRE2 microscope equipped with a Leica DFC 350FX camera and elaborated by a Leica FW4000 deconvolution software (Leica, Solms, Germany).

### RNA isolation, RT-PCR, and quantitative real-time RT-PCR

To generate cDNAs corresponding to the transcripts encoded by the target genes investigated extraction of total cellular RNA was performed using the Oligotex RNeasy Mini Kit (Qiagen, Hilden, Germany) and reverse-transcription with Superscript II (Invitrogen, Heidelberg, Germany). The integrity of the cDNAs generated was investigated by amplification of p53 transcripts, using the primers 5'-CGT GAG CGC TTC GAG ATG TCC G-3' (sense) and 5'-CCT AAC CAG CTG CCC AAC TGT AG-3' (antisense) as described previously [13]. For end point RT-PCR analysis of individual transcripts 0.5 μl first-strand cDNA were amplified using the QuantiTect SYBR Green PCR Kit (Qiagen), transcript-specific oligonucleotides (300 nM each) and 1U HotStarTaq DNA polymerase (Qiagen) in a 30 μl reaction, 40 cycles, in accordance with the manufacturer's instructions. Each PCR reaction was performed in triplicates using the following reaction conditions: initial denaturation/activation for 15 minutes at 95°C, 30 seconds at 94°C, 30 seconds of annealing, and 30 seconds at 72°C. A template-free negative control was included in each experiment. To amplify fragments corresponding to a defined *htid *splice-form (1,13), the following primer combinations were used: h*tid-L*, 5'-GTT GAC ATT CAA TCA AGC TGC-3'(sense) and 5'-CTG GGA TAT CAT GAG GTA AAC-3'(antisense), h*tid*-*I*, 5'-GTT GAC ATT CAA TCA AGC TGC-3'(sense) and 3'-CCA GTG GAT CTT TTT CCA GAG -3'(antisense) and h*tid*-*S*, 5'-CAG CCT CAG GAA GAA ACC ATC-3'(sense) und 5'-GGG ATC GTC ACG TTG ATC GTC-3' (antisense) according to reaction conditions as described previously [13]. For the amplification of an *HER-2 *[16] specific fragment, encompassing nt 1850-2157 of the corresponding cDNA (NCBI reference sequence NM_001005862.1; http://www.ncbi.nlm.nih.gov/sites/entrez) the primers 5'-CTC TGC TTC GTG CAC ACG GTG-3' (sense) and 5'-CAG GTC ACT GAG CCA TTC TGG-3' (antisense) (Eurofins MWG Operon, Ebersberg, Deutschland) were used at an annealing temperature of 60°C. The relative expression level (ΔCt) of a specific transcript was calculated with respect to the internal standard hypoxanthine guanine phosphoribosyl transferase (HGPRT) used in each reaction run to normalize variances in the quality of RNA, sample loading and the amount of input cDNA. Amplification of HGPRT was performed using the primers 5'-TGA CAC TGG CAA AAC AAT GCA-3' (sense) and 5'-GGT CCT TTT CAC CAG CAA GCT-3' (antisense) at an annealing temperature of 62°C. Quantitative real-time RT-PCR analysis was performed using the ABI PRISM 7300 Sequence Detection System instrument and software (Applied Biosystems). The analysis of relative target expression was performed using the 2^-ΔΔCt ^method [19]. To monitor DNA synthesis the fluorescent dye SYBR-Green was used. The cycle number at which the amplification of the transcript of interest was first detected is referred to as the cycle threshold, the Ct value. The increase in the fluorescence signal depends on the amount of the DNA in the starting PCR sample. The higher the DNA concentration, the faster a significant increase in fluorescence resulting in a low Ct value. The Ct value is proportional to the logarithm of the initial amount of the target DNA in the sample. The relative concentration of one target with respect to another is reflected in the difference of the cycle number, the ΔCt value. In this study the differences in the Ct values between the target gene/splice form investigated (X) and the reference (R, here HGPRT) are referred to as ΔCt values and were calculated as follows: ΔCt(X) = Ct(X) - Ct(R). A ΔCt = 0 indicates a ratio of 1 between the target and the reference (1 = 2^0 ^= 2^-(ΔCt)^). The factor 2 in the formula describes doubling of the fluorescence, the Ct value, at each cycle during the exponential phase of the PCR with 100% efficiency (E). Since E is determined by the RT-PCR conditions, the E values were optimized for each target gene investigated prior to the sample analysis. For the ΔCt calculation to be valid, the efficiency of amplification of both the target and the reference must be approximately equal. The E values determined were as follows: E_HGPRT _= 0,955; E_h*tid*-L _= 0,923; E_h*tid*-I _= 0,943; E_h*tid*-S _= 0,926; E_*erbB*-2/*her*-2 _= 0,906. The E values were calculated using the equation: E = 10^(-1/slope)^. The Ct values versus cDNA concentration input were plotted to calculate the slope (mean ± SD). Regressions were calculated using the graphad prism software as described previously [13]. The expression levels of the transcripts investigated in tumor samples were normalized to the respective normal tissues using ΔΔCt calculation as described previously [13]. The reported RNA expression levels represent the mean values (n = 3) ± standard deviation (SD). After standardization of both tumor and normal samples with respect to HGPRT, the change, ΔΔCt, in the expression levels of the target transcripts in the tumor sample as compared to normal was calculated as follows: ΔΔCt = ΔCt_tumor sample _ -ΔCt _normal sample_.

### Statistical examination

Statistical evaluation of the RT-PCR data was performed using the one-sided Student's t-test.

For the statistical analysis of the correlation of the expression of the two target antigens determined by immunohistochemistry the Fischer's exact test was employed.

## Results

### The expression of h*tid *and *HER-2 *RNA is altered in human breast tumors

The h*tid *tumor suppressor gene encodes three splice forms - h*tid-L*, h*tid-I *and h*tid-S *- generated by alternative splicing [1,7,13]. To determine the splice variant specific expression of the distinct h*tid *transcripts in normal breast epithelium and in breast cancers, we performed quantitative RT-PCR analysis (Table 1 and 2). In the context of the suppressive activity of hTid proteins on ErbB-2 [15] the samples were further investigated for the expression of the corresponding transcript. The evaluation of the amount of the targets investigated was performed using the comparative Ct/ΔΔCt method (cf. Materials and Methods). As shown in Table 1 in normal breast epithelium the three h*tid *splice forms are expressed at distinct levels. The 2^-ΔCt ^values (Table 1) indicate highest expression for the h*tid-I *form (2^-ΔCt ^= 0,50) and the lowest (2^-ΔCt ^= 3,18 × 10^-5^) for the h*tid-S *form. Generally, this expression profile corresponds to that we described previously for normal colon epithelium [13]. The expression level of the *HER-2 *transcript (2^-ΔCt ^= 4,99) in the breast epithelium samples which were defined as normal is higher (tenfold) as compared to the h*tid-I *RNA (Table 1). As shown in Table 2, all breast tumors investigated express aberrant RNA levels of all h*tid *splice forms as compared to normal tissue, independently from the level of the *HER-2 *transcript detected. With regard to the relative change of expression of the latter in the tumor samples as compared to normal the tumors can be divided into three groups. Whereas the first group is characterized by *HER-2 *levels ranging between 6-43% (samples 1-7) as compared to normal (100%), the third class, consisting of samples 14 and 15, shows drastic elevation (1048% and 1593%). The second group (samples 9-13) show slight to moderate increase of *HER-2 *expression ranging from 124% to 211%. One case, 8, shows similar *HER-2 *level as that detected in normal sample (Table 1 ). Comparing the TNMG status of the samples and the h*tid *expression profiles implicates that aspects of tumor progression such as tumor extension (T), lymph node affection (N) and loss of differentiation (G) are accompanied by elevation of h*tid*-L level (Table 2, cases: 3,7,8,12,13,15) ranging from 124 to 834%. In contrast, poorly differentiated and undifferentiated cases are characterized by decrease of h*tid*-L (Table 2, cases: 1, 4-6 and 11). With regard to the expression of the ht*id*-I form, the cases can be divided into two groups, those which show lower expression as compared to normal (Table 2, cases: 1-4, 7, 9, 11, 14, 15) and those with levels elevated up to twofold (Table 2, cases: 5, 6, 8, 10, 12 and 13). Similarly, h*tid*-S expression in the tumors is either down regulated in a range from 76% to 16%, (Table 2 , cases: 2-4, 9 und 14) or increased in a range from 114% to 380% (Table 2, cases: 1, 5-8, 10-13 and 15). With respect to the two latter forms, h*tid*-I and h*tid*-S, no correlation to the TNMG status is recognizable. Generally, the deregulation of the expression levels of the single h*tid *transcripts leads to a collapse of the concentration ratio among the single h*tid *forms and between the h*tid *variants and the *HER-2 *transcript.

**Table 1:** Quantification of the relative amounts of the three h*tid *splice forms *L*, *I *and *S*, and the *HER*-2 transcript in human normal breast epithelium

Target gene/splice form	*htid-L*	*htid-I*	*htid-S*	*erbB-2*
**Samples/ΔCt-value**	4,11	1,54	15,82	-2,75
	3,17	0,59	15,38	-1,96
	3,53	0,57	13,69	-2,26
	3,78	0,84	14,95	
	4,12	1,51	14,87	

**average ΔCt (=100%)**	**3,74**	**1,01**	**14,94**	**-2,32**

	± 0,3 (8,00%)	± 0,4 (39,61%)	± 0,5 (3,35%)	± 0,29 (12,50%)

**Amount of target:** **2^-ΔCt^**	**0,07**	**0,50**	**3.18 x 10^-5^**	**4,99**

The ΔCt value is determined by substracting the HGPRT (endogenous reference) Ct-value from the Ct value of the target investigated: ΔCt = Ct (target) - Ct (HGPRT); Standard deviation (SD): , n = 3, was calculated for the average ΔCt (100%); The relative concentration of the target (amount of target), 2^-ΔCt^, is calculated for the average ΔCt values.

**Table 2:** Relative expression of the h*tid *splice forms *L*, *I*, *S *and the *HER*-2 transcript in human breast cancer

Sample		1	2	3	4	5	6	7	8
T/N/M/G status		2/1/1/3	1/0/0/3	4/1/x/2	3/1/1/3	2/1/1/3	2/1/1/3	1/2/0/2	2/1/0/3
Target gene									
**h*tid-*L**	**ΔCt**	4,68	3,32	2,77	5,81	4,38	4.08	2,08	2,57
	**ΔΔCt**	0,94	-0,43	-0,98	2,07	0,64	0,34	-1,66	-1,18
	**2^-ΔΔ^Ct**	0,52	1,35	1,97	0,24	0,64	0,79	3,16	2,27
									
**Rel. expression (%)**		**52**	**135**	**197**	**24**	**64**	**79**	**316**	**227**

**h*tid-*I**	Δ**Ct**	1,55	2,99	2,21	2,89	0,77	0,82	1,88	0,35
	**ΔΔCt**	0,54	1,28	1,20	1,88	-0,24	-0,19	0,87	-0,66
	**2^-ΔΔ^Ct**	0,69	0,41	0,44	0,27	1,18	1,14	0,55	1,58
									
**Rel. expression (%)**		**69**	**41**	**44**	**27**	**118**	**114**	**55**	**158**

**h*tid-*S**	Δ**Ct**	14,55	15,61	15,34	15,84	13,39	13,43	14,75	14,22
	**ΔΔCt**	-0,4	0,67	0,4	0,90	-1,55	-1,51	-0,19	-0,72
	**2^--ΔΔ^Ct**	1,32	0,63	0,76	0,54	2,93	2,85	1,14	1,65
									
**Rel. expression (%)**		**132**	**63**	**76**	**54**	**293**	**285**	**114**	**165**

***HER-*2**	Δ**Ct**	1,63	0,44	0,29	-0,11	0,60	-0,69	-1,11	-2,37
	**ΔΔCt**	3,95	2,76	2,61	2,21	1,72	1,63	1,21	-0,05
	**2^-ΔΔ^Ct**	0,06	0,15	0,16	0,22	0,30	0,32	0,43	1,04
									
**Rel. expression (%)**		**6**	**15**	**16**	**22**	**30**	**32**	**43**	**104**

**Sample**		**9**	**10**	**11**	**12**	**13**	**14**	**15**	
									
**T/N/M/G status**		**2/0/0/2**	**1/x/x/2**	**1/2/0/3**	**3/1/x/3**	**4/2/0/3**	**4/2/0/3**	**3/4/0/3**	
									
**Target gene**									

**h*tid-*L**	Δ**Ct**	3,47	3,43	4,08	0,68	3,44	3,89	2,08	
	**ΔΔCt**	-0,28	-0,31	0,34	-3,06	-0,31	0,15	-1,66	
	**2^-ΔΔ^Ct**	1,21	1,24	0,79	8,34	1,24	0,90	3,16	
									
**Rel. expression (%)**		**121**	**124**	**79**	**834**	**124**	**90**	**316**	

**h*tid-*I**	Δ**Ct**	2,40	0,06	1,77	0,37	-0,08	0,90	1,28	
	**ΔΔCt**	1,39	-1,06	0,76	-0,64	-1,09	0,11	0,27	
	**2^-ΔΔ^Ct**	0,38	2,08	0,59	1,56	2,13	0,93	0,83	
									
**Rel. expression (%)**		**38**	**208**	**59**	**156**	**213**	**93**	**83**	

**h*tid-*S**	Δ**Ct**	15,91	13,02	13,90	13,84	13,32	17,55	13,51	
	**ΔΔCt**	0,97	-1,92	-1,04	-1,10	-1,62	2,61	-1,43	
	**2^-ΔΔ^Ct**	0,51	3,80	2,06	2,14	3,07	0,16	2,70	
									
**Rel. expression (%)**		**51**	**380**	**206**	**214**	**307**	**16**	**270**	

***HER-*2**	Δ**Ct**	-2,63	-2,97	-3,00	-3,23	-3,40	-5,71	-6,31	
	**ΔΔCt**	-0,31	-0,65	-0,68	0,91	-1,08	-3,39	-3,99	
	**2^-ΔΔ^Ct**	1,24	1,57	1,60	1,88	2,11	10,48	15.93	
									
**Rel. expression (%)**		**124**	**157**	**160**	**180**	**211**	**1048**	**1593**	

The samples are sorted according to the increase of *HER*-2 expression. The average ΔCt values (n=3) are presented. The calculation of ΔΔCt involves substraction by the ΔCt calibrator value (normal sample), ΔΔCt = ΔCt (tumor sample) - ΔCt (normal sample). The SD of ΔΔCt is the same as the SD of the ΔCt. The ΔCt values of both the tumor and normal samples are standardized to HGPRT used as endogenous control. The values defined as increase of expression represent the relative (rel.) change/increase in expression of the defined target as compared to normal sample and are calculated as follows: 2^-ΔΔCt ^× 100%. T, tumor extension: T1, to submucosa; T2, to muscle layer; T3, to subserosa; T4, to serosa. N, lymph node affection; M, metastasis; G, grading: G1, well differentiated; G2, moderately differentiated; G3, poorly differentiated; x, not defined.

### Expression of h*tid *in breast tumors as revealed by immunohistochemistry

**Figure 1:**
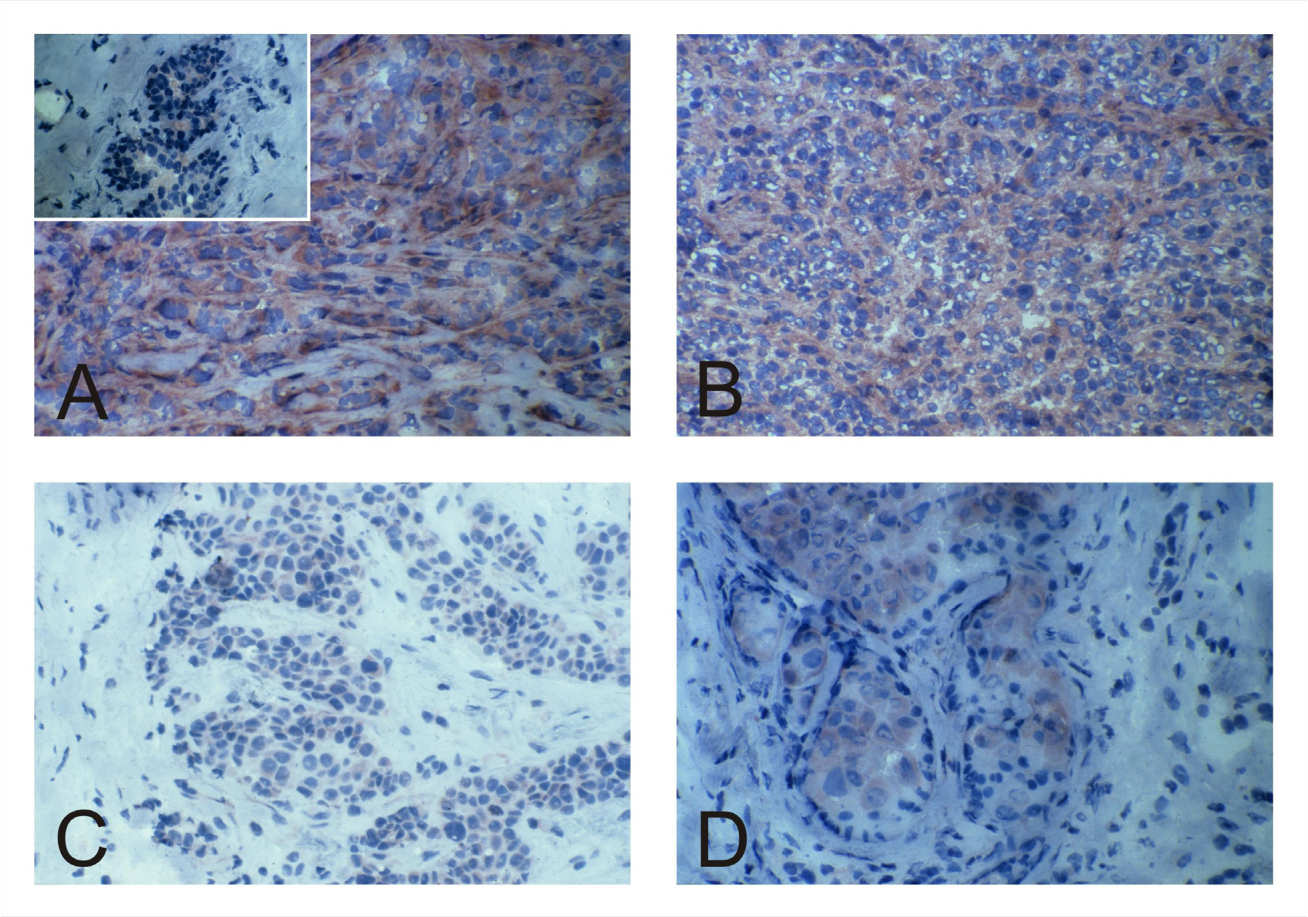
**Representative expression patterns of h*tid *in normal mammary epithelium and in primary breast tumors of different subtype**. Indirect immunoperoxidase staining using the Vectastain ABC Kit was performed according to manufacturer's suggestions using the polyclonal rabbit anti-hTid (6,7,13). Nuclei were counterstained with Mayer's hematoxylin. While h*tid *is expressed at low levels in the normal breast epithelium (A: insert) its expression is significantly elevated in the luminal A (A) and B (B) tumor type. In contrast, h*tid *is barely detectable in tumors of the HER-2 (C) and the triple negative (D) subtype. (Original magnification: 250x).

To obtain an overall assessment of h*tid *expression in normal and transformed mammary epithelium we submitted to immunohistochemical analysis morphologically normal breast tissue (3 cases), random selected infiltrating ductal carcinomas (75 cases) and metastatic lesions (30 cases). Staining of these substrates with the affinity purified hTid antiserum [6,7,13] demonstrated focal expression of the chaperone molecule only in the epithelial component of morphologically normal breast (Figure 1A, insert). In contrast about 43% of primary and 30% of metastatic tumors (including lymphonodal and extra-nodal lesions) were characterized by positive staining ranging from moderate to intense staining.

In the context of the potential association of h*tid *expression with the biology of human breast cancers of different subtype the immunohistochemical analysis revealed elevated h*tid *expression in 81% of the luminal A type tumors (Figure 1A), in 20% of the cases of the luminal B type over expressing ErbB-2 and in 18% of tumors diagnosed as the HER-2 subtype (not shown). Tumors of the triple negative type showed positive staining for anti-Tid in 40% of the cases investigated (Figure 1B-D).

### Correlation between h*tid *and ErbB-2 expression in breast and non-breast tumors over expressing the ErbB-2 receptor as revealed by immunohistochemistry

**Figure 2:**
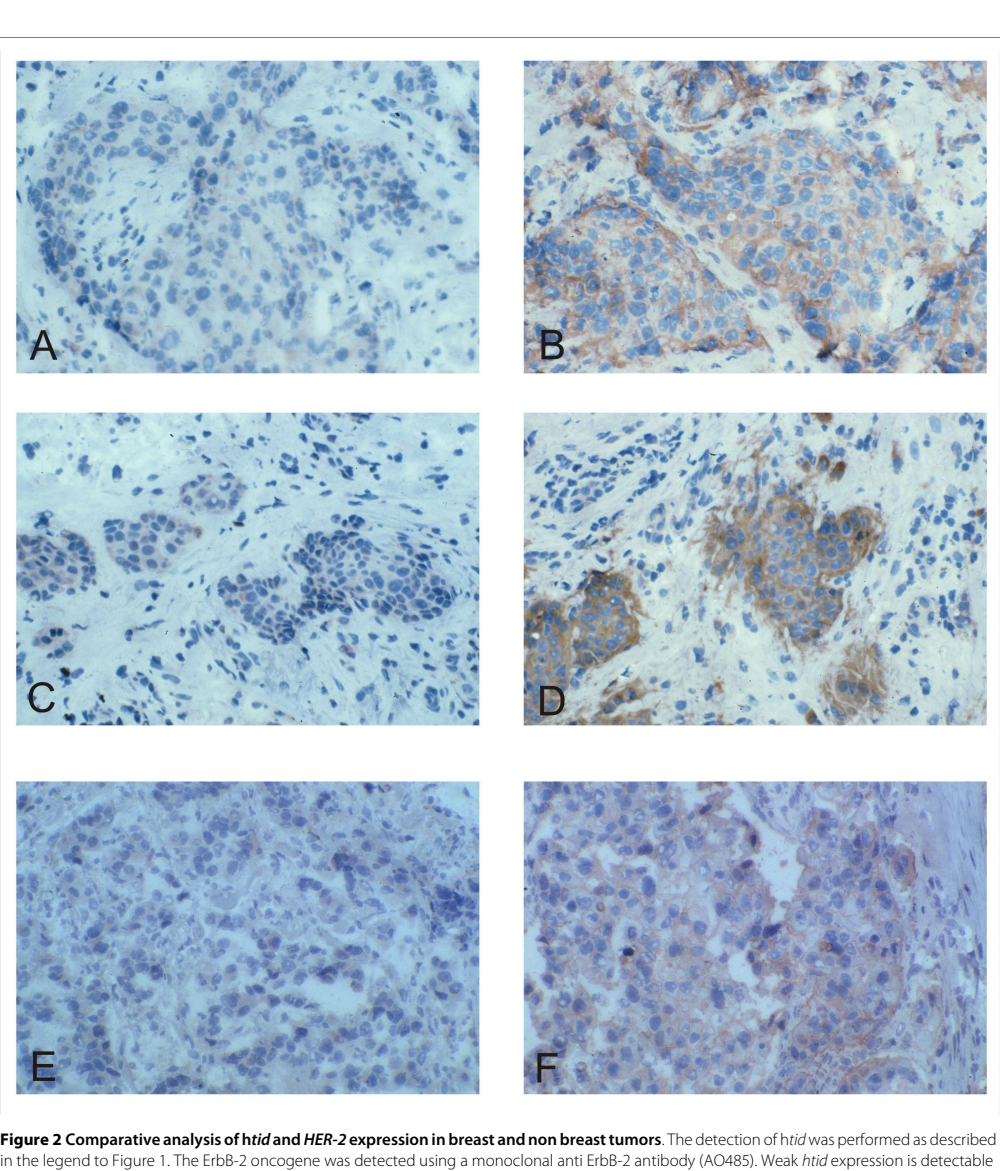
**Comparative analysis of h*tid *and *HER-2 *expression in breast and non breast tumors**. The detection of h*tid *was performed as described in the legend to Figure 1. The ErbB-2 oncogene was detected using a monoclonal anti ErbB-2 antibody (AO485). Weak *htid *expression is detectable in *HER-2 *over expressing breast tumors of the luminal B (A, B) and HER-2 (C, D) subtype as well as in a renal clear cell carcinoma (E, F). (Original magnification: 250×).

In view of the above findings which are consistent with the in vitro findings of hTid oncosuppressive activity on ErbB-2 [15], we focussed our further investigation on the analysis of the in vivo correlation between h*tid *and *HER-2 *expression. Regarding the expression of the two target genes the following patterns were compared: double positive (*HER-2 *+/*tid *+), double negative (*HER*-2 -/*tid *-) and positive/negative (*HER-2 *+/*tid *-; *tid *+/*HER-2 *-). Specimens characterized by lack or faint expression of the target antigens were defined as negative. Samples showing moderate (+) to strong (2+/3+) signals by staining them with anti hTid antibodies and 3+ or 2+/Fish+ stain using antibodies against ErbB-2 were defined as positive. In that context we assayed comparatively the expression levels of the proteins encoded by the two target genes in 24 primary tumors over expressing the receptor (17 of the HER-2 and 7 of the luminal B subtype) and 34 ErbB-2 negative tumors (16 luminal A, 8 luminal B and 10 of the triple negative subtype) as well as in metastatic lesions over expressing the TRK receptor by staining with anti-Tid [6,7,13] and antibodies against ErbB-2. The results of this analysis are summarized in Table 3A and Figure 2A-D. They clearly demonstrate that the expression of the two targets investigated is inversely correlated in both primary (p < in 0,0001) and in metastatic (p < 0.023) mammary tumors (not shown). Since ErbB-2 over expression may occur also in other epithelial cancers [18], we performed a comparative staining of *HER-2 *and h*tid *in non mammary carcinomas characterized by elevated levels of ErbB-2 (10 cases) and in cancers displaying low levels of the receptor (8 cases). This study (Table 3B) also yielded a highly significant inverse correlation (p < 0,007) with respect to the expression levels of the two tumor genes investigated (Figure 2E, F).

**Table 3:** The expression of hTid and ErbB-2 is inversely correlated in primary breast cancer (A) and in non mammary tumors (B).

A^1^	hTid (+)	hTid (-)	B^2^	hTid (+)	hTid (-)
ErbB-2 (+)	2*	22*	ErbB-2 (+)	2*	8*

ErbB-2 (-)	28*	6*	ErbB-2 (-)	8*	0*

*: number of cases; ^1^: p< 0,0001; ^2^: p< 0,007;

### Correlation between h*tid *and ErbB-2 expression in breast tumors induced in *Her-2/neu *transgenic mice

**Figure 3:**
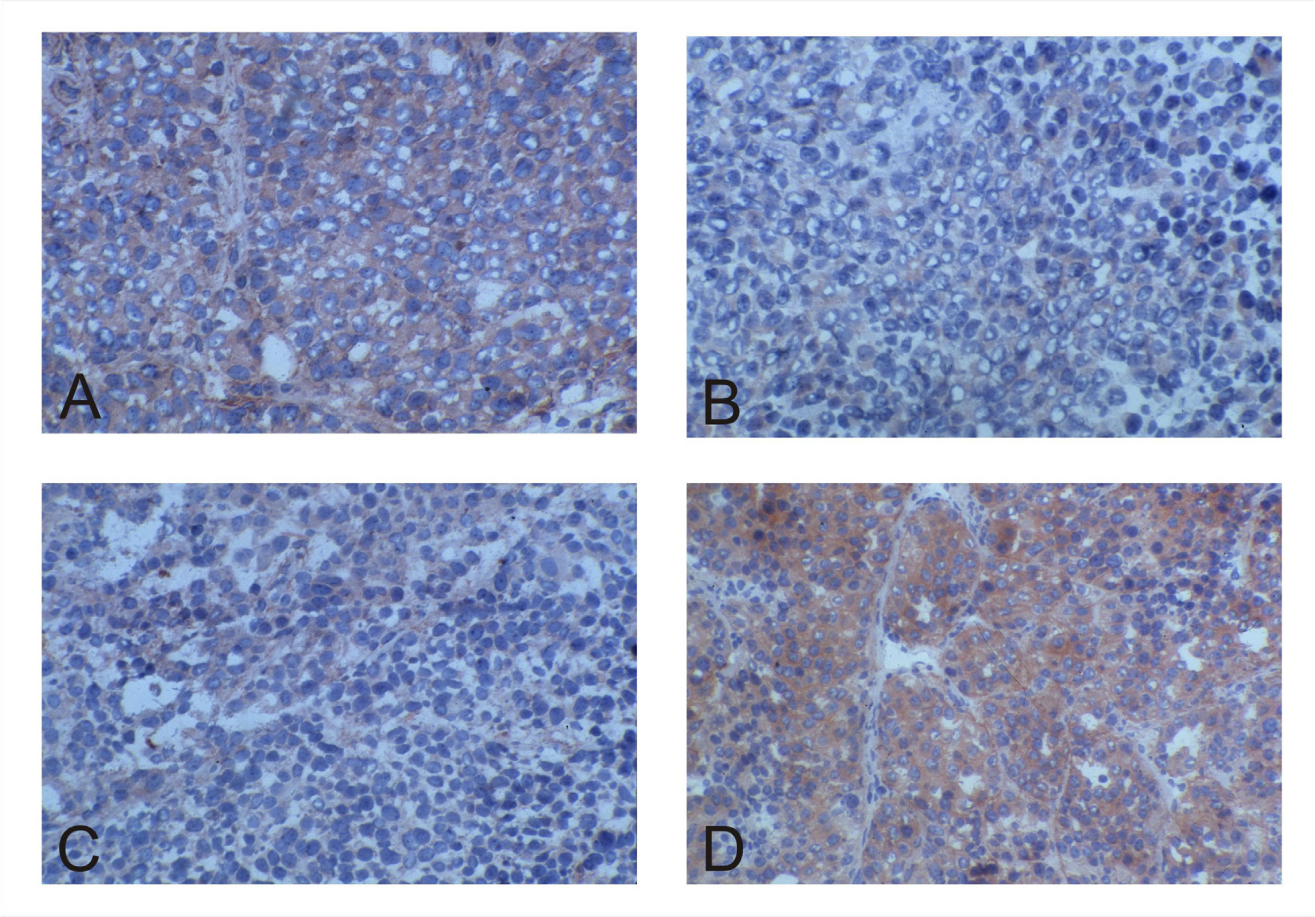
**Comparative analysis of h*tid *and *HER-2/neu *expression in breast tumors arising in transgenic Balb/c mice carrying the rat *HER-2/neu *oncogene revealed inverse correlation between the expression levels of the investigated targets**. The detection of m*tid *was performed as described in the legend to Figure 1. ErbB-2 was detected using the monoclonal anti ErbB-2 antibody (AO85) (cf. Figure 2) cross reacting with the rat molecule. While early tumors, 22 week old, display high *tid *(A) and low *HER-2/neu *levels (B), those harvested at 30 weeks are characterized by a low *tid *expression (C) in contrast to high *HER-2/neu *(D) levels. (Original magnification: 250×).

**Figure 4:**
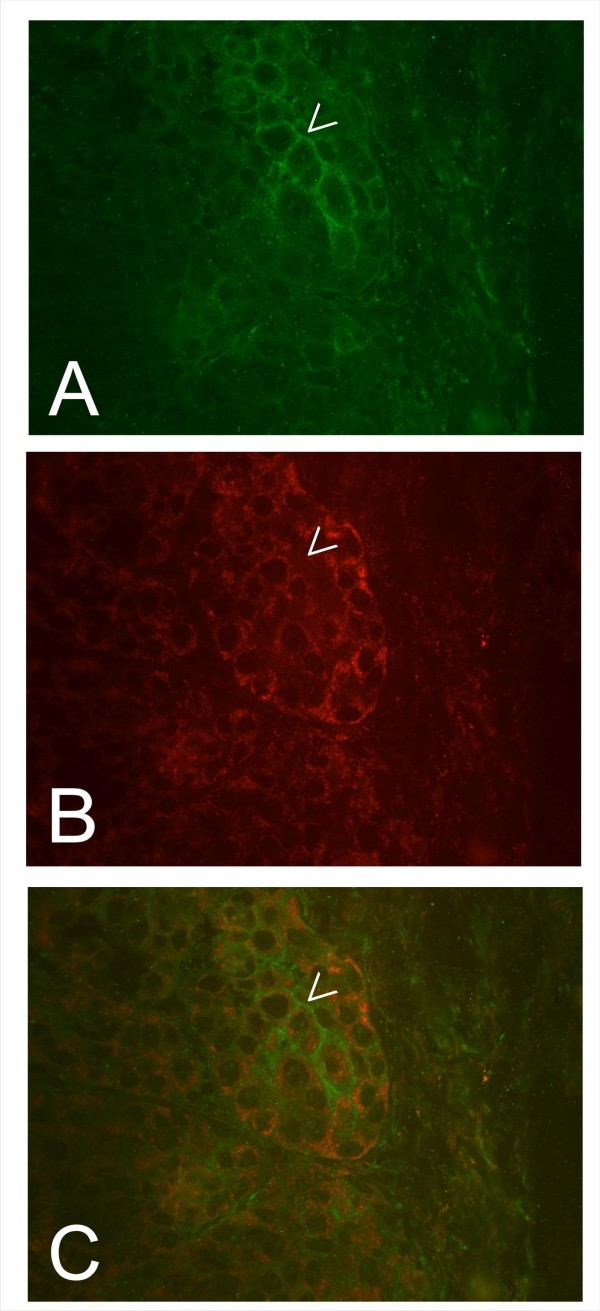
**Photographs of simultaneous staining of 30 week old breast tumors arising in *HER-2/neu *transgenic mice illustrating that over expression of *HER-2/neu *correlates down regulation of h*tid***. ErbB-2 was detected using a polyclonal chicken anti rat ErbB-2 and a FITC labelled secondary antibody. Tid was detected using the rabbit polyclonal anti hTid antibody [6,7,13] and a Texas Red labelled secondary antibody. The tumor cells over expressing ErbB-2 (A, arrowhead) are characterized by low *tid *expression (B, arrowhead; cf. Figure 3). In C an overlay of the photographs shown in A and B is presented.

As described above, the inverse correlation of the expression profiles of the two proteins in human breast tumors and non mammary malignancies provides an in vivo proof for the oncosuppressive activity of h*tid *on ErbB-2 described in vitro [15]. Furthermore, since the inverse relationship is highly significant in diverse tissues, this functional link is not cell specific, and therefore of general biological importance. Next, we asked whether the inverse correlation is evolutionarily conserved in the mouse. To answer this question we employed the transgenic mice model of *Her-2/neu *induced breast tumors [23]. We stained early (22 weeks) and late (30 weeks) breast tumors generated in transgenic mice carrying the rat *Her*-2/*neu *oncogene. As shown in Figure 3A, staining of the early tumors with the hTid [7,13] antiserum revealed homogenous (1+) cytoplasmic expression. Differently, using the anti-ErbB-2 antiserum cross reacting with the murine receptor resulted in a faint stain of the cytoplasm and rarely in staining of the membrane of the tumor cells (Figure 3B). In the more advanced 30 weeks tumors the staining patterns changed with regard to both the expression level and distribution. In these lesions, the hTid expression was heterogeneous with alternating areas of moderate to intense (2+) cytoplasmic stain (Figure 3C). The expression of ErbB-2 also appeared heterogenous with only discrete areas of the tumor displaying an intense (2+) staining often cell membrane associated (Figure 3D). This result further suggested that the inverse correlation of the expression levels of the two molecules is indeed detectable also in experimental tumors raised in mice carrying the rat *Her-2/neu *oncogene. In order to conclusively prove this issue, we submitted the more advanced tumors to double staining using anti-hTid [5,13] and the chicken antibody recognizing the murine TRK receptor molecule. As shown in Figure 4, tumor areas characterized by intense membrane ErbB-2 expression (**A**) display significantly lower h*tid *level (**B, C**), thus, demonstrating the inverse correlation of the expression of the two tumor relevant molecules and implying their functional link.

## Discussion

The understanding of the mechanism responsible for the in vivo oncosuppressive action of h*tid *in human tumorigenesis is of biological relevance in view of the multiple molecular interactions of the proteins it encodes [7-15,25,26]. The ability of the hTid proteins to interact with distinct cancer related molecules, mediating via linked signal transduction networks diverse cellular processes, suggests that the deregulation of their expression may affect simultaneously diverse cellular functions. Furthermore, the identification of the h*tid *encoded proteins as components of multi-component complexes suggests that their activity is likely to be associated with mechanisms determining the sequential and temporarily determined assembling of these complexes in the cell and their cellular topology. This is consistent with the function of molecules defined as chaperones and their molecular assistants known as co-chaperons. The Tid proteins [1,2,6,7,13] are indeed members of the DNAJA3 family encoding Hsp70/Hsc70 co-chaperone molecules [4,5]. The understanding of their role in the spatial and temporal organization of either active or silent protein complexes is of relevance in the context of both general developmental processes and, especially, tumor biology [6-15]. Regarding the latter the knowledge of the biological context these molecules are involved in may have an essential impact for the identification of novel causal cancer therapies.

Data are available indicating that the cytosolic hTid-proteins L and I mediate cellular tumor-related processes such as proliferation, differentiation and migration by modulating/stabilizing signalling pathways driving these processes [6,7,9-14]. Generally, two modes of action of the Tid proteins can be discerned to date: i) interaction with cytosolic molecules, such as the APC tumor suppressor [7,13], a central component of the Wingless/Wnt pathway and the E-Cadherin mediated signalling or the Inhibitor of IKB in the NFκB signalling [11], crucial for driving the expression of regulators of cell cycle control, or ii) binding to receptors mediating the activation of signalling pathways, e.g. Ptc [6,7] and ErbB-2 [15]. With regard to the latter the regulatory functions of the cytosolic h*tid *splice forms hTid-L and hTid-I have been shown to participate in the degradation of the receptor mediated by the Hsp70/CHIP ubiquitin ligase complex [15]. Interestingly enough, both hTid forms, L and I, are suggested to be equally capable to down regulate the over expression of the ErbB-2 receptor and to decrease its oncogenic signalling in human breast cancer cell lines in which the co-chaperone molecule was over expressed [15]. This observation strongly suggests that the ErbB-2 concentration level is relevant in activating the suppressive function of the two hTid proteins [15]. Furthermore, it raises the question whether the binding of the two hTid forms with ErbB-2 is functionally equal also under physiological circumstances. We asked these questions during the study presented here. Functional equality under physiological conditions suggests, as a result, that these two proteins may complement each others' function(s) under peculiar circumstances, e.g. pathological states.

Overall, the in vivo data of the present study further support the growing evidence that the anticancer activity of the hTid proteins occurs through the interference with multiple oncogenic pathways by binding and modifying functions of key molecules maintaining intracellular signalling. The ErbB-2 TRK represents one of these molecules.

As revealed by the RT-PCR analysis, the three h*tid *splice forms are differentially expressed in normal breast epithelium. Their concentration levels are in this tissue similar to those we described previously for colon epithelium [13], the I form with the highest expression level and the S form characterized by very low concentration. This suggests that under normal physiological conditions the concentration of the three h*tid *splice forms is precisely regulated and that this mechanism is essential for the diverse functions the single molecules are maintaining in the cell. Previously we showed that the expression profile changes in epithelial tumors such as basal cell carcinomas (BCCs) [6] and colon cancers [13]. As shown here, this is also true for human breast tumors which are characterized by alteration of expression of all three h*tid *forms resulting at least in a collapse of the concentration ratio between them. Since the hTid proteins are highly promiscuous [6-15] this phenomenon must, as a result, drastically affect the homeostasis of the cellular functions these complexes are involved in. Thus, the deregulation of h*tid *leads independently of the causative event to severe cellular derangements. Additionally to this general conclusion a further more specific observation, concerning the aforementioned in vivo functional equality of the two forms L and I, can be driven from the present study. The RT-PCR data demonstrate up-regulation of expression of the hTid-L form in most of the non metastasizing tumors, whereas poorly differentiated and undifferentiated cases with lymph node metastases show a decrease of the level of this form up to 24% of the normal (100%) level. This pattern which suggests that hTid may exert oncosuppressive activity which breaks down during tumor progression is consistent with the role postulated for the L and I forms [15] by in vitro studies employing the ErbB-2 over expressing cell lines. Furthermore, the RT-PCR data indicate that the hTid-L oncosuppressive action may be of general importance being not limited to ErbB-2 over expressing malignancies. In view of this, one can hypothesize at the present the following model for the activation of this security/emergency mechanism: if the concentration of a molecule driving the tumorigenic process, e.g. ErbB-2, riches a threshold level hTid-L expression is enhanced. This rescuing mechanism however is time limited as hTid expression declines during tumor progression. This hypothesis raises questions concerning the mechanisms regulating the physiological and emergency expression of h*tid*. The answer to this question is the subject of our present investigations. So far we can state that consistent with the molecular promiscuity of the hTid proteins distinct cell specific and cell status dependent regulatory mechanisms are involved in these processes. As previously mentioned, the functional equality of L and I under physiological circumstances would suggest that these two forms may complement each others function under specific circumstances. Under the assumption that this is true a shift of the concentration ratio of the two forms could be expected as a consequence of their oncosuppressive/protective function on the tumorigenic targets. Though this hypothesis is from the mechanistic point of view very attractive, the analysis of the correlation of the expression levels of the two forms L and I in the tumor samples do not support it at present. Decrease in the level of the L form do not correspond to increase of the concentration of the I form and vice versa. Thus, to solve the problem of the functional equality of the two hTid forms L and I further investigations using adequate in vivo methodology are necessary. With regard to the h*tid *splice forms I and S, which expression levels are also changed in nearly all tumor samples investigated, no correlation with the TNMG status and the *HER-2 *level could be found.

The immunohistochemical analysis described in this study provided additional interesting findings. It showed that the changes in the expression profiles of the targets investigated during tumor progression of experimental tumors are not ubiquitous but rather hot-spot like. This is consistent with the fact that functional complexes including the Tid proteins are built in the cells sequentially and are topologically determined as we discussed previously [6,7]. Most importantly, the data provide in vivo evidence that h*tid *is a potential modulator of ErbB*-2 *signalling in both breast and non-breast tumors over expressing the kinase. Furthermore, since the inverse correlation of the expression levels of the two genes is highly significant in human breast cancers as well as in experimental breast tumors the functional relationship between the two targets is conserved. Furthermore, the transgenic mice animal model may be suitable to in vivo dissect the oncosuppressive activity of the hTid proteins on ErbB-2 function. In view of the association of ErbB-2 over expression with poor prognosis in breast cancers the elucidation of these phenomena is of particular biological interest also in the context of the pharmacological relevance of ErbB-2 regulation, which, possibly, could be modulated via tuning of expression of the single h*tid *splice forms. With regard to the latter reduction of h*tid *expression can in fact render cancer cells resistant to apoptosis induced by a wide spectrum of stimuli including TNF-α [25] and increase their migratory potential [26].

The variety of biological processes the EGFR family members regulate results from the differential signalling they mediate by building both homo and diverse heterodimers between the family members responding to distinct ligands [16,27-29]. ErbB-2 is the favourite partner of all other EGFR receptors [29]. The ErbB-2/ErbB-3 heterodimer is a high affinity receptor for neuregulins endowed with potent oncogenic activity [28]. In view of this the hTid proteins can also be considered as potential EGFR and/or HER-3 binding partners and therefore players in the signalling they induce. In this context a preliminary comparative expression analysis of hTid, EGFR and ErbB-3 we have conducted in triple negative breast tumors and in cutaneous melanoma (data not shown) respectively also suggests an inverse correlation between the expression levels of hTid and the two receptors, thus, indicating that h*tid *functional relation may indeed encompass other members of the EGFR family. Since these receptors control different developmental processes their activation/deactivation requires a fine tuning. The on status is activated by paracrine and autocrine signals engaging one or more receptors [30]. The off status is strengthened by a number of feedback inhibitors [30]. So far four ErbB-2 inhibitors have been described, namely, LRIG1 [31], SOCSS4 and 5 [32] and RALT/MIG6 [33,34]. While the first three inhibitors interfere temporary with EGFR activity by enhancing its ubiquitination, RALT is endowed with the ability to block the ErbB RTK signalling by its inception [33]. Our in vivo results suggest hTid-L as a further modulator of ErbB-2 activity in human tumors of epithelial origin. In accordance with the reported molecular analysis in vitro [15] it can be postulated that this may occur via mechanisms similar to those described for the negative feedback inhibitors LRIG1 and SOCS4 and 5. This hypothesis has to be confirmed using appropriate experimental approaches.

## Conclusions

In summary, the presented data provide in vivo evidence for h*tid *function as a negative regulator of ErbB-2 activity. The inverse correlation of the expression levels of the two genes is highly significant in human breast and non-mammary cancers, and in experimental tumors raised in transgenic mice carrying the rat *HER-2/neu *oncogene. Thus, the functional relationship between the two targets is tissue independent and evolutionarily conserved.

## Competing interests

The authors declare that they have no competing interests.

## Authors' contributions

UKD designed and evaluated the RT-PCR experiments, generated and purified the anti-hTid antibody and drafted the manuscript. MH determined h*tid *expression via RT-PCR. MRN performed the immunohistochemistry of human and murine carcinomas. MK determined *HER-2 *expression via RT-PCR. PGN designed and analyzed the immunohistochemical data and drafted the manuscript. All authors read and approved the final manuscript.
